# Comparison between Percutaneous Gastrostomy and Self-Expandable Metal Stent Insertion for the Treatment of Malignant Esophageal Obstruction, after Propensity Score Matching

**DOI:** 10.3390/nu12092756

**Published:** 2020-09-10

**Authors:** Joo Hye Song, Jaehyun Ko, Yang Won Min, Kyunga Kim, Hyuk Lee, Byung-Hoon Min, Jun Haeng Lee, Poong-Lyul Rhee, Jae J. Kim

**Affiliations:** 1 Department of Medicine, Samsung Medical Center, Sungkyunkwan University School of Medicine, Seoul 06351, Korea; joohye.song@samsung.com (J.H.S.); jaehyun0820.ko@samsung.com (J.K.); lhyuk.lee@samsung.com (H.L.); jason.min@samsung.com (B.-H.M.); jh2145.lee@samsung.com (J.H.L.); pl.rhee@samsung.com (P.-L.R.); jaej.kim@samsung.com (J.J.K.); 2 Statistics and Data Center, Research Institute for Future Medicine, Samsung Medical Center, Seoul 06351, Korea; kyunga.j.kim@samsung.com

**Keywords:** esophageal neoplasm, self-expandable metallic stents (SEMS), gastrostomy, enteral nutrition, survival

## Abstract

Background: The outcomes of the two procedures; self-expandable metal stent (SEMS) insertion and percutaneous gastrostomy (PG) feeding procedures, used in patients with malignant esophageal obstruction, are still controversial. We aimed to compare the outcomes between the two procedures, following propensity score (PS) matching. Methods: We retrospectively reviewed 568 esophageal cancer patients who underwent SEMS insertion (stent group) or PG (gastrostomy group) at the Samsung Medical Center between January 1996 and December 2018. Procedures for reasons other than malignant obstruction were excluded. We analyzed the datasets after PS matching. Primary outcomes were the post-procedural nutritional status, and need for additional intervention (AI). The secondary outcome was overall survival (OS). Results: In a matched cohort, the gastrostomy group showed less decrease in albumin level after the procedure (−0.15 ± 0.57 vs. stent group; 0.41 ± 0.59, *p* = 0.021). The gastrostomy group required less need for, and number of, AIs (2.1% vs. stent group; 23.4%, *p* < 0.001 and 0.04 ± 0.25 vs. stent group; 0.31 ± 0.61, *p* < 0.001). After matching, there was no significant difference between the two groups in OS. However, PG was associated with OS based on multivariable analysis of the matched cohort (vs. stent group, hazard ratio 0.69, 95% confidence interval 0.5–0.95). Conclusions: PG tends to provide better post-procedure nutritional status than SEMS insertion in patients with malignant esophageal obstruction.

## 1. Introduction

Esophageal cancer is the eighth most common cancer and sixth leading cause of cancer-related mortality worldwide [1]. More than 50% of cases of esophageal cancer are usually diagnosed at an advanced stage, and dysphagia is the most common symptom, which contributes to weight loss and malnutrition [2]. Self-expandable metal stent (SEMS) insertion was established as the standard treatment for patients with malignant esophageal obstruction [3]. However, SEMS insertion led to adverse outcomes, such as chest pain, fistula, and stent migration [4,5]. Percutaneous gastrostomy (PG) feeding was established as an alternative treatment for malignant esophageal obstruction, and several studies suggested that PG could provide stable nutritional status and a better quality of life (QoL) compared to stent insertion [6,7,8,9,10,11].

From a physiological point of view, stent insertion is an ideal procedure [4,12]. However, there were inconsistent outcomes and a lack of strong evidence supporting its implementation for optimal treatment of patients with malignant esophageal obstruction. Usually, multidisciplinary evaluation before deciding on the route of feeding is required [13]. When the management of malignant esophageal obstruction is planned by a physician, it is important to predict the nutritional outcomes after the procedure is conducted. This is mainly because esophageal cancer exhibits a high risk of malnutrition related to cancer cachexia and dysphagia [2].

Therefore, in our study, we aimed to compare the outcomes related to nutritional and survival benefits between SEMS insertion and PG feeding for patients with malignant esophageal obstruction by applying the propensity score matching method.

## 2. Materials and Methods

### 2.1. Study Population

**Figure 1:**
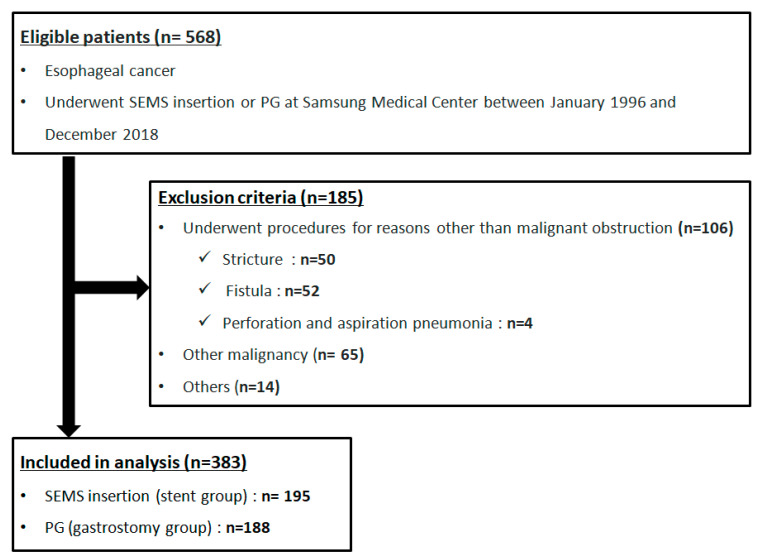
Flow chart of the study population. SEMS, self-expandable metal stent; PG, percutaneous gastrostomy.

We retrospectively reviewed 568 esophageal cancer patients who underwent fully covered SEMS insertion (stent) or PG (gastrostomy) at the Samsung Medical Center between January 1996 and December 2018. Exclusion criteria were as follows: (1) underwent procedures for reasons other than malignant obstruction (*n* = 106), such as stricture (*n* = 50) (due to radiation therapy (*n* = 12), endoscopic submucosal dissection (*n* = 8), or esophagectomy (*n* = 30)), fistula (*n* = 52), esophageal perforation (*n* = 1), and aspiration pneumonia (*n* = 3); (2) coexisting other malignancies (*n* = 65); and (3) others (*n* = 14), such as underwent the procedure previously at another hospital (*n* = 7), recurrent cancer after radiotherapy and esophagectomy (*n* = 2), lye stricture (*n* = 1), underwent jejunostomy prior to the procedure (*n* = 1), did not follow-up (*n* = 1), and tissue type of esophageal cancer was unclear or unclassified (*n* = 2). Finally, a total number of 383 patients, including 195 patients who underwent SEMS insertion and 188 patients who received PG feeding, were considered for further analysis in this study (Figure 1).

On 15 May 2020, the Institutional Review Board of the Samsung Medical Center provided their approval to conduct this study (2020-05-018-001). The study protocol conformed to the ethical guidelines of the 1975 Declaration of Helsinki, as reflected in a prior approval by the institution’s human research committee. The requirement for informed consent from patients was waived because only de-identified data routinely collected during hospital visits were used.

### 2.2. Indication of Procedure

At our hospital, difficulty in providing sufficient nutrition due to dysphagia was considered as an indication for performing either SEMS insertion or PG. When the attending physician chose the procedure type (stent or gastrostomy), the physician discussed with patients the known benefits and complications of each procedure based on general clinical experience. And finally, the physician decided the route for feeding. In case of cervical esophageal obstruction, the physician preferred PG to stent insertion due to the difficulty in maintaining the position of the stent. Conversely, when it was difficult to perform PG due to anatomical reasons, the physician preferred stent to PG. Endoscopic stent insertion was performed under conscious sedation using endoscopy. Once the obstruction was adequately delineated and guide wire access through the entire length of the obstruction obtained, the SEMS was advanced across the obstruction and deployed. The stent was deployed under a combination of endoscopic and fluoroscopic guidance. Oral feeding was possible from the day after stent insertion. PG was also performed under endoscopic (percutaneous endoscopic gastrostomy, PEG) or fluoroscopic guidance (percutaneous radiologic gastrostomy, PRG). Prophylactic intravenous antibiotics were administrated before the procedure. The PEG tube was inserted by the pull method. The puncture site was marked with endoscopic monitoring of the anterior gastric wall in the lower body by trans-illumination, and, following adequate local anesthesia, an appropriate initial incision was made and the puncture cannula was inserted under endoscopic control through the stomach. The PRG tube was inserted directly through the abdominal wall into the stomach by the push method. Enteral feeding was possible 24 h after the procedure, only if the patient had no abdominal pain and displayed normal bowel sound. About 50 mL of normal saline/hour was administrated three times, and then 50 mL of semi-fluid diet feeding (SFD) was administered. The SFD feeding reached 250 mL by gradual increases in the amount of feeding, and then soft blend diet feeding was initiated.

### 2.3. Outcome Measurement

Baseline characteristics were assessed retrospectively by reviewing electronic medical records as follows: age at diagnosis, gender, tumor stage according to the 7th edition of the American Joint Committee on Cancer, tumor histology, tumor location, length of obstruction by tumor (assessed by esophagography, endoscopy or computed tomography (CT)), history of chemotherapy, radiotherapy, and esophagectomy before the procedure [14]. We also assessed the procedure-related adverse events (such as tumor bleeding, fistula, perforation, and chest pain in the stent group, and PG site infection, peritonitis, and leakage in the gastrostomy group), the presence and number of additional interventions (AIs) (including stent insertion/repositioning, gastrostomy or removal of stent/gastrostomy due to adverse events), and the occurrence of aspiration pneumonia. We assessed the presence of chest pain by reviewing medical records where the patient had subjectively complained of chest pain, or the physician had carried out a procedure for chest pain. Aspiration pneumonia was defined as the combination of a history of aspiration according to the medical records and gravity-dependent opacity in a chest CT scan after the procedure. Furthermore, in order to evaluate the post-procedural nutritional status, body weight and the serum albumin level at baseline and 1 month after the procedure were assessed.

Primary outcomes were post-procedural nutritional status (a change in serum albumin level and body weight between baseline and 1 month after procedure), the need for and number of AIs, and the occurrence of procedure-related adverse events and aspiration pneumonia. The secondary outcome was overall survival (OS).

### 2.4. Statistical Analysis

Continuous variables are expressed as mean ± standard deviation, while categorical variables are presented as absolute values and percentages. Differences between continuous variables were analyzed using the unpaired Student’s *t*-test and Wilcoxon rank sum test. The differences between categorical variables were analyzed using the χ2 test and Fisher’s exact test, accordingly. The propensity scores were estimated for age, gender, stage, length of obstruction and treatment before procedure using the parsimonious logistic regression model. The 1:1 matching without replacement was performed within 25% of standard deviation of log-transformed propensity scores, therefore the matched data were analyzed with exactly the same methods that can be used for the original data [15,16]. In the propensity score-matched cohort, the two groups were compared for the baseline characteristics, and the absolute standardized mean differences of variables were <0.2 to be balanced between two groups. OS was calculated using the Kaplan–Meier method and compared using the log-rank test. Cox hazard proportional models were used to examine the association of baseline characteristics with overall survival in the propensity score-matched cohort. Variables with a *p*-value < 0.2 in univariable analysis were later subjected to multivariable analysis. Differences with a *p*-value < 0.05 were considered statistically significant. All statistical analyses were performed using SPSS software version 25.0 for Windows (SPSS Inc., Chicago, IL, USA) and open source statistical language and platform, R, version 3.6.1 (R Development Core Team, Vienna, Austria), using the package “Matching.”

## 3. Results

### 3.1. Baseline Characteristics of the Study Population

Baseline characteristics of patients with malignant esophageal obstruction who underwent stent insertion and gastrostomy are shown in Table 1. Before matching, the patients in the stent group (*n* = 195) were identified to be in a more advanced stage, with a lower number of cervical cancer cases, and received chemotherapy, radiotherapy, and esophagectomy less often than the patients in the gastrostomy group (*n* = 188) (*p* < 0.001 for all the characteristics). After propensity score matching, there was no significant difference between the stent and gastrostomy groups (Table 2).

### 3.2. Primary Outcomes of the Propensity Score-Matched Cohort

Primary outcomes in the matched cohort are shown in Table 3. A total of 14 procedure-related adverse events occurred. In the stent group, there were 6 adverse events; stent broken (*n* = 1), stent migration (*n* = 2), stent induced tracheal compression (*n* = 1), tumor bleeding (*n* = 1), and chest pain (*n* = 1). In the gastrostomy group, there were 8 adverse events; leakage (*n* = 1), gastrostomy site infection (*n* = 4), and peritonitis (*n* = 3). The gastrostomy group showed less decrease in serum albumin level and needed less additional interventions (AIs) than the stent group after the procedure (*p*-value 0.021 and <0.001, respectively). In the stent group, AIs included stent reposition/removal due to migration, stent removal due to chest pain, and gastrostomy due to tumor ingrowth into stent. In the gastrostomy group, AIs included gastrostomy tube removal due to localized infection and gastrostomy revision due to leakage. The number of AIs was lower in the gastrostomy group than in the stent group (0.04 ± 0.25 vs. 0.31 ± 0.61, *p*-value < 0.001). There was no significant difference between the two groups with regard to body weight change, occurrence of procedure-related adverse events, and aspiration pneumonia.

### 3.3. Overall Survival of the Study Population

**Figure 2:**
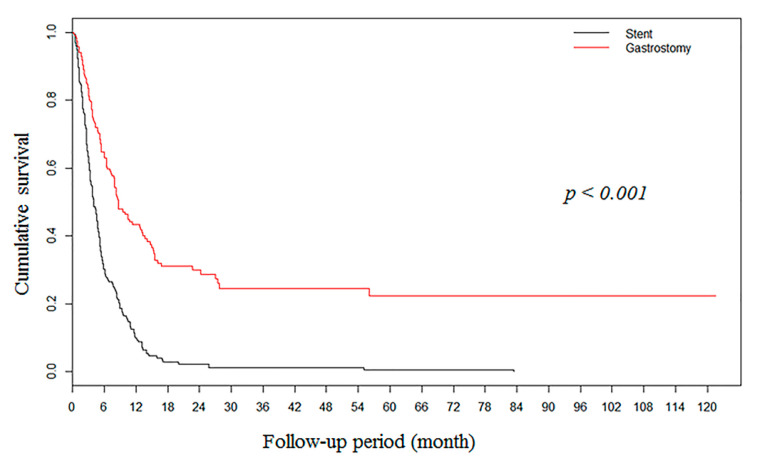
Kaplan–Meier survival curves showing overall survival rates of 383 patients with malignant esophageal obstruction after gastrostomy (*n* = 188) and stent insertion (*n* = 195).

**Figure 3:**
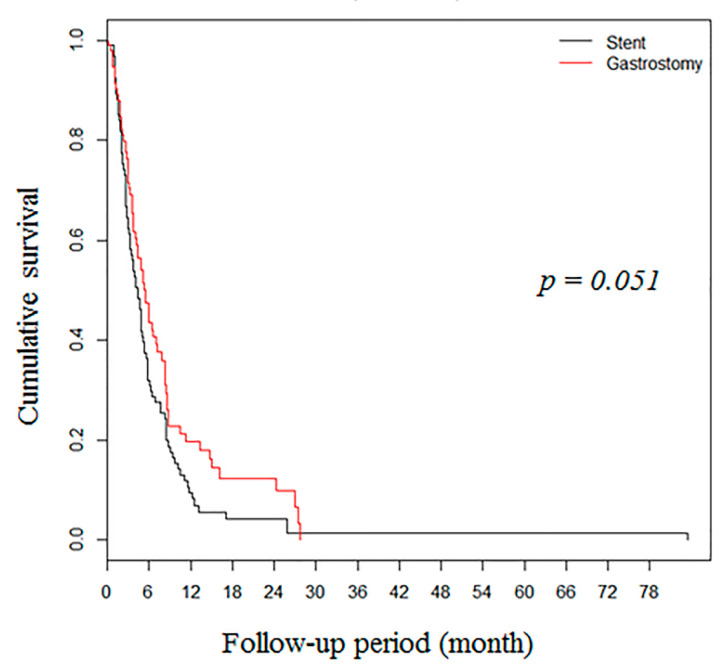
Kaplan–Meier survival curves showing overall survival rates in the propensity score-matched cohort (94 matched pairs) with patients with malignant esophageal obstruction after gastrostomy and stent insertion.

During median follow-up of 5 months (2.6–9.3), the gastrostomy group showed higher OS rates in the unmatched cohort (*n* = 383, Figure 2). However, after propensity score matching, there was no significant difference between the two groups (94 pairs) in OS (Figure 3).

### 3.4. Factors Associated with Overall Survival in Propensity Score-Matched Cohort

To adjust for potential confounders after propensity score matching, Cox proportional hazards regression model was conducted. Multivariable analysis showed that chemotherapy after procedure, surgery before or after procedure, and type of procedure (gastrostomy) were the independent factors associated with OS (Table 4).

## 4. Discussion

Currently, patients with malignant esophageal obstruction are either subjected to gastrostomy or SEMS insertion. It is mainly because the maintenance of nutritional status through these procedures provides better clinical outcomes, including improvement of QoL and survival benefits [17,18]. However, it has not been established yet whether these are the optimal treatment strategies. In our study, we demonstrated that PG was superior to SEMS, by assessing the patients’ nutritional status using propensity score matching analysis within a large cohort.

Systematic review and meta-analysis of nine studies comprising 180 patients showed that stent insertion relieved dysphagia immediately and led to an increase in both body weight and serum albumin level, but chest pain and stent migration occurred with high incidence rates (51.4% and 32%, respectively) [4]. A retrospective study with propensity score matching conducted by Mariette et al. (2015) found that SEMS insertion was a predictor of poor prognosis with adjusted confounding factors (hazard ratio (HR) 1.6, 95% confidence interval (CI) 1.02–2.5), even though the median time of recurrence and three-year survival were found to be reduced in the SEMS group [19]. Based on the above-mentioned two studies, the European Society of Gastrointestinal Endoscopy does not recommend the temporary placement of an SEMS/SEPS (self-expandable plastic stent) for malignant dysphagia as a bridge to surgery or before pre-operative chemoradiotherapy, and suggests other options, such as placement of a feeding tube (strong recommendation based on low-quality evidence) [3,13]. In our study, 93% (162/181) of patients in the stent group and 67% (129/188) in the PG group underwent procedure in palliative care. Because several cases were performed before current ESGE guidelines had been established, some patients underwent SEMS between late 1990 and early 2010 for non-palliative care. However, SEMS insertion was performed recently in our hospital only for palliative care, consistent with current ESGE guidelines. In our previous retrospective study, we first compared the two procedures, and then observed that PG feeding was associated with better OS in patients with malignant esophageal obstruction compared to SEMS insertion by stabilizing the nutritional status (HR 0.56, 95% CI 0.36–0.87) [20]. However, our previous study had several limitations that could be attributed to a relatively small sample size and risk of bias. To minimize the risk of bias, in the present study, we increased the sample size and analyzed the cohort thoroughly with propensity score matching and the obtained results were taken into consideration for further interpretation. Thereafter, we observed that gastrostomy resulted in less decrease in the serum albumin level compared to the stent group, after the procedure. This parameter reflected the nutritional status, and consequently, better outcomes. Indeed, in the present study, we showed that PG was an independent factor associated with OS (HR 0.69, 95% CI 0.50–0.95). Although there was no significant difference in OS between the two groups (*p* = 0.051), it was still very close to statistical significance. In contrast to other studies, there was no difference in procedure-related adverse outcomes, including chest pain, between the two groups. It could be explained by the assumption that at our hospital, PG was performed in cases of cervical malignant esophageal obstruction, which later resulted in chest pain due to stent insertion. Additionally, according to a recent prospective study conducted by Yu et al. (2018) comparing nasogastric tube, stent, and ostomy tube feeding, stent led to a poor QoL when compared with the other two groups [21]. On comprehensive evaluation, gastrostomy tended to be superior to SEMS in general aspect, including nutritional status and QoL.

In multivariable analysis, surgery after procedure showed the best OS, followed by only procedure without surgery, and surgery before procedure. Compared to patients who underwent only procedure without surgery, the risk of mortality was higher for those who underwent surgery before procedure (HR 123.08, 95% CI 10.15–1492.60). However, only one patient underwent surgery before procedure and the range of 95% CI was very wide. Considering these points, the risk was overestimated and it should be interpreted carefully.

Our study had some limitations. The first limitation is that it is a retrospective study. However, as many previous studies have demonstrated the potential advantages of gastrostomy, it was difficult to conduct a randomized controlled trial (RCT) in an ethical manner. Additionally, conducting prospective RCT to compare SEMS insertion and gastrostomy for the treatment of malignant esophageal obstruction was practically impossible. Instead, to obtain a high level of evidence close to a RCT, we first analyzed the study population thoroughly with propensity score matching, and then interpreted the results that were obtained. Second, because of the limitation of conducting a retrospective study, QoL was not assessed, which was recently identified as an important part of palliative treatment in patients with malignant esophageal obstruction [10]. Instead of error-prone symptom evaluation by retrospective analysis, we decided to assess objective parameters, such as body weight and serum albumin levels, as primary outcomes. Third, nutritional status was determined one month after the procedure, which might be too short a time for decreased body stores to be evidenced by weight loss and a decreased albumin level. Also albumin, an acute phase reactant, could not be used as a good nutritional marker. However, due to high mortality and poor general condition related to the high risk of malnutrition, there were a considerable number of missing data regarding body weight and albumin, more than two months after procedure; yet several studies have used albumin inevitably as a nutritional marker. Unavoidably, we assessed procedural nutritional status as a change of serum albumin level and body weight between baseline and one month after procedure. Fourth, esophageal squamous cell carcinoma is more common in Asian than Western populations, and only three esophageal adenocarcinoma cases were included in our study. So, this study may be unclear when generalizing the results to Western patients with malignant esophageal obstruction. Despite these limitations, we believe that we have provided enough evidence to support the advantage of PG through a well-designed analysis using propensity score matching.

## 5. Conclusions

In conclusion, we suggest that gastrostomy may be preferred over stent insertion in patients with malignant esophageal obstruction, considering the nutritional and survival benefits.

## Figures and Tables

**Table 1:** Comparison of baseline characteristics between patients with malignant esophageal obstruction undergoing gastrostomy and stent insertion, before propensity score matching (*n* = 383).

		Gastrostomy Group (*n* = 188)	Stent Group (*n* = 195)	*p*-Value
Age	year	63.80 ± 9.57	64.71 ± 10.36	0.37
Gender	Male	161 (85.6)	180 (92.3)	0.054
	Female	27 (14.4)	15 (7.7)	
Stage	Stage II + III	119 (63.3)	33 (16.9)	<0.001
	Stage IV	69 (36.7)	162 (83.1)	
Location	Cervical	50 (26.6)	5 (2.6)	<0.001
	Upper	54 (28.7)	37 (19.0)	
	Mid	41 (21.8)	68 (34.9)	
	Lower	43 (22.9)	85 (43.6)	
Histology	Squamous cell carcinoma	185 (98.4)	181 (92.8)	0.087
	Others	3 (1.6)	14 (7.2)	
Length of obstruction	cm	6.72 ± 3.17	6.53 ± 2.70	0.85
Chemotherapy	None	27 (14.4)	49 (25.1)	<0.001
	Before procedure	71 (37.8)	100 (51.3)	
	After procedure	90 (47.9)	46 (23.6)	
Radiotherapy	None	46 (24.5)	115 (59.0)	<0.001
	Before procedure	60 (31.9)	42 (21.5)	
	After procedure	82 (43.6)	38 (19.5)	
Surgery	None	130 (69.1)	187 (95.9)	<0.001
	Before procedure	1 (0.5)	0 (0.0)	
	After procedure	57 (30.3)	8 (4.1)	

**Table 2:** Baseline characteristics of patients with malignant esophageal obstruction undergoing gastrostomy and stent insertion, after propensity score matching (94 matched pairs).

		Gastrostomy Group (*n* = 94)	Stent Group (*n* = 94)	SMD
Age	year	64.67 ± 9.66	65.36 ± 10.05	0.070
Gender	Male	84 (89.4)	86 (91.5)	0.072
	Female	10 (10.6)	8 (8.5)	
Stage	Stage II + III	37 (39.4)	33 (35.1)	0.088
	Stage IV	57 (60.6)	61 (64.9)	
Location	Cervical	8 (8.5)	5 (5.3)	−0.13
	Upper	29 (30.9)	28 (29.8)	−0.023
	Mid	28 (29.8)	28 (29.8)	0.00
	Lower	29 (30.9)	33 (35.1)	0.091
Histology	Squamous cell carcinoma	92 (97.9)	93 (98.9)	0.085
	Others	2 (2.1)	1 (1.1)	
Length of obstruction	cm	7.06 ± 3.32	6.75 ± 2.93	0.098
Chemotherapy	None	20 (21.3)	23 (24.5)	0.076
	Before procedure	42 (44.7)	40 (42.6)	−0.043
	After procedure	32 (34.0)	31 (33.0)	−0.023
Radiotherapy	None	40 (42.6)	42 (44.7)	0.043
	Before procedure	28 (29.8)	29 (30.9)	0.023
	After procedure	26 (27.7)	23 (24.5)	−0.073
Surgery	None	89 (94.7)	88 (93.6)	−0.045
	Before procedure	1 (1.1)	0 (0.0)	−0.15
	After procedure	4 (4.3)	6 (6.4)	0.095

SMD, standardized mean difference.

**Table 3:** Comparison of primary outcomes between patients with malignant esophageal obstruction undergoing gastrostomy and stent insertion, in the propensity score matched cohort (94 matched pairs).

		Gastrostomy Group (*n* = 94)	Stent Group (*n* = 94)	*p*-Value
Weight change ^1^	kg	−0.69 ±2.56	−0.27 ± 3.48	0.58
Albumin change ^1^	g/dL	−0.15 ± 0.57	−0.41 ± 0.59	0.021
Additional intervention	None	92 (97.9)	72 (76.6)	<0.001
	Yes	2 (2.1)	22 (23.4)	
Number of additional interventions		0.04 ± 0.25	0.31 ± 0.61	<0.001
Procedure-related complications	None	90 (95.7)	91 (96.8)	1.00
	Yes	4 (4.3)	3 (3.2)	
Aspiration pneumonia	None	81 (86.2)	81 (86.2)	1.00
	Yes	13 (13.8)	13 (13.8)	

^1^ After procedure.

**Table 4:** Factors associated with overall survival in propensity score matched cohort with malignant esophageal obstruction (94 matched pairs).

		Multivariable Analysis	
		HR (95% CI)	*p*-Value
Stage	Stage II + III	1	
	Stage IV	1.43 (1.00–2.06)	0.052
Location	Cervical	1	0.30
	Upper	1.22 (0.64–2.34)	0.54
	Mid	1.63 (0.84–3.16)	0.15
	Lower	1.15 (0.59–2.23)	0.69
Chemotherapy	None	1	0.072
	Before procedure	0.89 (0.57–1.39)	0.62
	After procedure	0.60 (0.38–0.95)	0.029
Radiotherapy	None	1	0.031
	Before procedure	1.55 (1.00–2.39)	0.050
	After procedure	0.79 (0.51–1.22)	0.29
Surgery	None	1	<0.001
	Before procedure	123.08 (10.15–1492.60)	<0.001
	After procedure	0.34 (0.14–0.82)	0.016
Type of procedure	Stent	1	
	Gastrostomy	0.69 (0.50–0.95)	0.024

HR, hazard ratio; CI, confidence interval.
